# A High-Risk Patient: Chronic Care Refusal and Severe Constipation Before Medical Aid in Dying

**DOI:** 10.7759/cureus.113355

**Published:** 2026-07-25

**Authors:** Karen R Morin

**Affiliations:** 1 Hospice, End-of-Life Care, and Medical Aid in Dying Services, LA Patient Advocates, Los Angeles, USA

**Keywords:** adult malnutrition, bloodstream absorbtion, bowel disfunction, end of life and hospice care, gastro-paresis, medical aid in dying, patient autonomy, psychology, refusal of standard treatment, supportive and palliative care

## Abstract

In jurisdictions where medical aid in dying is legally available to qualified terminally ill patients, certain clinically observed physiologic characteristics are associated with prolonged times to death following the ingestion of prescribed medications. But patients’ behavioral factors may also contribute to this risk.

This report highlights how prolonged refusal of medical care, resulting in malnutrition and immobility, contributed to a high-risk clinical profile and an unusually prolonged time to death following medication ingestion.

## Introduction

Medical-aid-in-dying protocols emphasize thorough patient assessments to minimize the incidence of prolonged or distressing deaths following medication ingestion. Present aid-in-dying medication protocols use a combination of digoxin, diazepam, morphine, amitriptyline, and phenobarbital (DDMAPh). The medications must be self-administered by the patient into the gastrointestinal tract; injections (intravenous, intramuscular, other) are forbidden by aid-in-dying laws. Although the patient may be terminally ill, a functional gastrointestinal tract is essential for the absorption of the medications. The Academy of Aid-in-Dying Medicine lists specific baseline clinical factors known to increase the likelihood of delayed medication absorption or unpredictable physiologic responses, particularly with oral aid-in-dying regimens [1]. Behavioral patterns may exacerbate those risk factors and increase the likelihood of longer or more complicated assisted deaths, yet they have been less thoroughly described [2].

This paper describes a patient who declined all medical interventions, leading to a terminal condition and hospice care. She then requested medical aid in dying and was eligible due to her terminal illness and capacity for medical decision-making. Her course was complicated by psychosocial complexity intersecting with physical factors known to be associated with prolonged or complicated aid-in-dying deaths.

## Case presentation

A 66-year-old female experienced advanced functional decline following an occlusive stroke resulting in a left hemiparesis. She required high levels of assistance with all activities of daily living. Nonetheless, her lack of mobility and resistance to help led to prolonged times in bed. A defining feature of this patient’s history was a lifelong pattern of refusing medical care, including pain management and bowel regimens. Over several years, she developed multiple untreated skin malignancies with ulcerating lesions, some complicated by maggots, involving her chest and extremities, accompanied by recurrent infections. She reported a pattern of approximately one bowel movement per month, associated with significant pain and distress. She continued to refuse all bowel hygiene aids.

Based on social characteristics and refusal of care, the patient’s oral intake was limited to minimal fluids only. Clinical appearance showed severe cachexia, and, combined with dietary history, was consistent with severe malnutrition.

The patient consistently refused all recommended interventions, including stool softeners, laxatives, prokinetics, suppositories, enemas, hydration strategies, and dietary modification. Despite these findings, she remained cognitively intact, alert, and fully oriented, with no evidence of delirium, depression, or impaired decision-making capacity as defined in standard clinical assessments [3]. Her preferences were consistent over time, reflecting a deeply held aversion to medical intervention even in the setting of significant discomfort.

The patient was referred to the author at LA Patient Advocates by her hospice after she requested “death with dignity.” Her hospice does not facilitate medical aid in dying; instead, it refers patients to outside clinicians. The referral noted that the patient had a long history of refusing medical interventions, but she was clear that she wanted to use medical aid in dying. The author spoke with her nephew, with whom she lived, and explained her role as a nurse who facilitates the aid-in-dying process. She scheduled the first telemedicine call with the prescribing physician and made plans to be at their home for the call.

It was not until meeting the patient in her home that the extent of her refusal of all medical interventions became clear. As noted above, her clinical appearance demonstrated severe cachexia despite a distended abdomen and absent bowel sounds. Abdominal palpation demonstrated probable stool. The patient stated no bowel movement in the past 15 days. Rectal exam was deferred. Vital signs were within normal limits. The patient complained of severe abdominal pain and declined all pain medications. Her husband had died eight years previously, at a young age, after a misdiagnosis, precipitating the patient’s refusal of all types of medical interventions, no matter how small.

The patient’s nephew cared for her at home, with a hospital bed in her bedroom. Three years prior, during her hospitalization for the stroke with hemiparesis, multiple skin cancers and infections were discovered, including an open wound with maggots.

The author/nurse educated the patient and her nephew about the processes for medical aid in dying. She emphasized the possible effects the patient’s long-standing constipation would have on the absorption of the end-of-life medication, and urged laxatives and enemas, consistent with her usual recommendation of frequent bowel movements preceding aid in dying. The patient refused all suggestions.

Rectal administration of the aid-in-dying medications was discussed, explaining that this route would avoid her dysfunctional bowel. She remained adamantly against any interventions and decided to take the medications orally. She had no vomiting or nausea. The patient was a woman of few words, but she made her wishes quite clear.

In preparation for the aid-in-dying day, the author stated directly to the patient and her nephew that a very long time to death was anticipated, but assured them that she would fall asleep and be deeply unconscious in a short time, usually 5 to 10 minutes. Support for the nephew on that day was also discussed.

The patient chose a weekend day for her death, for the convenience of her nephew. On that day, the author again explained that a full and dysfunctional bowel would delay the absorption of the medications, possibly prolonging her time in a coma before dying. The patient again refused all bowel preparations. The hospice nurse and social worker were both present for support, but, per their policy, left before the ingestion.

The author mixed the DDMAPh powders with 2 ounces of water (DDMAPh: digoxin 100 mg, diazepam 1 g, morphine 15 g, amitriptyline 8 g, phenobarbital 5 g) [4]. The bottle of suspended powders was handed to the patient, and she drank it with a milkshake straw. She was unconscious within five minutes. The nephew was reassured that his aunt was not suffering and was in a coma. Breathing was monitored, and the author waited with the nephew for a little over 2.5 hours, in case she died within the commonly expected time.

When she was still alive after that, the nephew was reassured that awakenings with DDMAPh had never been reported and that his aunt would die, though the timing was uncertain. The author left the house but maintained hourly contact for the next five hours. That evening, the patient was still breathing. The nephew called the author the following morning to say that his aunt had died 31 hours after taking the aid-in-dying medications.

## Discussion

This patient highlights the intersection of physiologic and behavioral characteristics that may increase the risk of a prolonged or complicated aid in dying. Severe untreated constipation, malnutrition, chronic infection, immobility, and the consistent refusal of supportive interventions, particularly with bowel care, substantially increased this patient’s risk of an extremely long time to death. Of note, the patient was unconscious and comfortable throughout the 31 hours. The hospice staff, per policy, left the home before the patient took the medications. During the 31-hour wait, the nephew needed repeated reassurance that his aunt was no longer suffering and that awakenings had not been reported with DDMAPh [5,6]. The reasons for this patient’s extremely prolonged time to death were multiple. Severe chronic constipation raises concerns for impaired gastrointestinal motility, including possible gastroparesis with delayed gastric emptying and slowed transit of orally administered medications to the duodenum, where most drug absorption occurs [7]. This was complicated by long-term malnutrition with likely global gut wasting and diffuse loss of peristaltic and absorptive function. Interestingly, her time to sleep was five minutes, within a typical range, which speaks to how even her minimally functioning gut readily absorbed phenobarbital. Malnutrition may further affect drug distribution and metabolism, increasing pharmacokinetic variability [8,9]. Additional physiologic stressors, including her chronic infection, systemic inflammation, immobility, and advanced debility, may have prolonged time to death following the oral ingestion of aid-in-dying medications by disrupting expected pharmacokinetic and pharmacodynamic processes.

Inflammatory states and immobility are also associated with delayed gastric emptying and impaired intestinal absorption, potentially postponing attainment of plasma drug concentrations necessary for rapid sedation and coma [10]. Concurrently, infection-related inflammation may alter hepatic enzyme activity and metabolic capacity, resulting in unpredictable drug effects and delayed progression from sedation to respiratory depression and cardiac arrest [8].

When a patient demonstrates such an elevated risk for an extremely prolonged aid-in-dying death, clinicians must provide full informed consent for the procedure [11,12,13]. Such patients should be informed that they will remain unconscious and comfortable even during prolonged aid-in-dying deaths, but the lengthy process may be extremely stressful for loved ones in attendance. And while awakenings have not been reported with DDMAPh [5,6], we can never exclude that very rare possibility in such high-risk patients. Clinicians can consider refusing the option of aid in dying while offering assertive palliative care for a comfortable death. Review by an ethics consultation service might be considered [14]. For this patient, the risk was not considered high enough to refuse aid in dying. But for all extremely risky patients, clinicians must provide patients, families, and clinical staff with thorough explanations of the risks, benefits, and alternatives before proceeding.

Future explorations of patients with extreme malnutrition/cachexia should ascertain whether the rectal route (which does not require peristaltic function) might work better than the oral route. Careful explanations of both routes and why one over the other might be indicated for the outcome are important. The concept of global gut dysfunction preceding aid in dying is a relatively new consideration, and increasing our knowledge of the variations of this disorder will be essential. The role of high-dose DDMAPh for these high-risk patients should also be considered [4].

## Conclusions

Patients with a lifelong history of refusing medical and symptomatic treatments may be at increased risk for prolonged or unpredictable outcomes of medical aid in dying. Clinicians should provide ongoing education and support for the patient and family or significant others. Integration with hospice care is essential.

This case underscores the importance of assessing not only medical diagnoses but also long-standing behavioral patterns when evaluating patients for medical aid in dying.
